# 

**Published:** 1842-01

**Authors:** 


### CONTENTS

OF NO. I.

### ORIGINAL COMMUNICATIONS.

### ESSAYS AND CASES.

ART. I.—A Report of cases treated at the Louisville Marine Hospital. By RICHARD W. FERGUSON, M. D. 1
ART. II.—A case of Labour, attended by some unusual appearances. By JOHN OVERTON, M. D., of Nashville, Tenn. - - - - - 15
ART. III.—Observations on the Topography, Climate, and Diseases of a District in the Eastern part of the State of Ohio. By THOMAS CARROLL, M. D., late of St. Clairsville, now of Cincinnati, Ohio. - - 20
ART. IV.—A case of injury of the Head with loss of a portion of the Brain and recovery. By DR. THO. H. TODD, of Starkville, Mississippi. - - - - 39
ART. V.—Case of Chronic Cystitis, terminating fatally. By Dr. R. B. HARPER, of Tipton county, Tennessee. 41
ART. VI.—A Description of the different grades of Bilious Remittent Fever, as they occur in the Wabash Valley, and particularly in Clark county, Illinois. By JAMES SMICK, of Darwin, Illinois. - - - 42
ART. VII.—Remarks on a Review of an Essay on Trembles, Milk-sickness, &c. By JOHN S. SEATON, M. D. 64

Art. VIII.—Singular Phenomena accompanying Menstruation and Uterine Hemorrhage. By Dr. J. E. Sands, of Wilson county, Tennessee. - - - 69

SELECTIONS FROM AMERICAN AND FOREIGN JOURNALS.

Rheumatism of the Skin. - - - - - 71
Poisoning from the use of Tobacco Enema. - - 72
Conium and Opium. - - - - - 73
Report on the Treatment of Burns, &c. - - - 74
Extract of Cicuta in Gonorrhœa. - - - - 76
Use of Quinine in Dropsy. - - - - - 76
On the condition of the nails in fractured limbs. - - 77
Extract of Belladonna in Paraphimosis. - - - 77

ORIGINAL INTELLIGENCE.

Editorial Department. - - - - - 79
Kentucky State Medical Society. - - - - 79
Lithotomy. . - - - - - - 80
Physiological Temperance Society. - - - - 80
Alleged discharge of hairs from the hand. - - - 80

